# Blood leukocytes from benznidazole-treated indeterminate chagas disease patients display an overall type-1-modulated cytokine profile upon short-term in vitro stimulation with *trypanosoma cruzi* antigens

**DOI:** 10.1186/1471-2334-12-123

**Published:** 2012-05-24

**Authors:** Renato Sathler-Avelar, Danielle Marquete Vitelli-Avelar, Silvana Maria Elói-Santos, Eliane Dias Gontijo, Andréa Teixeira-Carvalho, Olindo Assis Martins-Filho

**Affiliations:** 1 Laboratório de Biomarcadores de Diagnóstico e Monitoração, CPqRR-FIOCRUZ, Belo Horizonte, MG, Brazil; 2 Unicentro Newton Paiva, Belo Horizonte, MG, Brazil; 3 Universidade Vale do Rio Verde, UninCor, Belo Horizonte, MG, Brazil; 4 Departamento de Medicina Preventiva e Social, Faculdade de Medicina, Universidade Federal de Minas Gerais, Belo Horizonte, Minas Gerais, Brazil; 5 Departamento de Propedêutica Complementar, Faculdade de Medicina, Universidade Federal de Minas Gerais, Belo Horizonte, Minas Gerais, Brazil; 6 Laboratório de Biomarcadores de Diagnóstico e Monitoração, R, Laboratório de Biomarcadores de Diagnóstico e Monitoração, CPqRR-FIOCRUZ, Av. Augusto de Lima 1715, 30190–002, Belo Horizonte, MG, Brazil

**Keywords:** Chagas disease, Benznidazole, Immune response, Cytokines, Leucocytes subsets, CNPq, FAPEMIG, FIOCRUZ

## Abstract

**Background:**

Benznidazole (Bz)-chemotherapy is recommended to prevent Chagas disease progression, despite its limited efficacy during chronic disease. However, the host mechanisms underlying these benefits still remain to be elucidated.

**Methods:**

In this study, we have used short-term whole blood cultures to describe the cytokine profile of Bz-treated Indeterminate Chagas disease patients-(INDt) as compared to untreated patients-(IND).

**Results:**

Our findings showed that IND presented increased levels of IL-10^+^neutrophils, IL-12^+^ and IL-10^+^monocytes and IFN-γ^+^NK-cells. Moreover, IND showed slight increase of IL-4^+^CD4^+^T-cells and enhanced levels of IL-10^+^CD8^+^T-cells and B-cells. Additional analysis of cytokine Low and High producers also highlighted the presence of High cytokine producers within IND, including IL-10 from CD4+ T-cells and IFN-γ from CD8^+^ T-cells, as compared to NI. The Bz-treatment lead to an overall cytokine down-regulation in the innate and adaptive compartments, including low levels of IL-12^+^ and IL-10^+^neutrophils and monocytes, IFN-γ^+^NK-cells, IL-12^+^, TNF-α^+^, IFN-γ^+^ and IL-5^+^CD4^+^T-cells and IL-10^+^B-cells, along with basal levels of cytokine-expressing CD8^+^T-cells in INDt as compared to IND. The in vitro antigen stimulation shifted the cytokine profile toward a type 1-modulated profile, with increased levels of IL-12^+^ and IL-10^+^ monocytes, IFN-γ^+^ and IL-4^+^NK-cells along with TNF-α^+^ and IFN-γ^+^CD8^+^T-cells. Analysis of Low and High cytokine producers, upon in vitro antigen stimulation, further confirm these data.

**Conclusion:**

Together, our findings showed that the Bz treatment of Indeterminate Chagas’ disease patients shifts the cytokine patterns of peripheral blood monocytes, NK-cells and CD8^+^ T-cells towards a long-lasting Type-1-modulated profile that could be important to the maintenance of a non-deleterious immunological microenvironment.

## Background

An outstanding triumph of the Brazilian medicine was the discovery of Chagas disease by Carlos Chagas at the beginning of the 20^th^ century. One century after the discovery, several aspects of Chagas disease still remain unclear, including those regarding to the multiple events of pathogenesis underlying the clinical progression, as well as the mechanisms implicated in the successful etiological treatment.

Chagas disease is caused by the haemoflagellate protozoan *Trypanosoma cruzi* and is considered one of the most important public health problems in Latin America, affecting approximately 8 to 10 million people and a further 100 million people are considered at risk of infection [1].

The major mechanism associated with Chagas disease pathology is the continuous inflammatory infiltrate observed mainly in the cardiac and digestive tract organs characterized by damages in the neuronal conductive systems and also by muscle cytolysis [2]. There is a growing consensus that complex co-adaptation between the persistent parasite infection and the balanced host immune response underlies the establishment of Indeterminate clinical form of Chagas disease. On the hand the persistence of parasite along with unbalanced immune response leads to a sustained inflammatory response that supports these characteristic lesions of chronic Chagas disease [2]. In this immunopathogenesis paradigm the eradication of *T. cruzi* may be a pre-requisite to control the disease progression and prevent the irreversible long-term consequence of Chagas disease. Therefore, Chagas disease must be treated primarily as an infectious disease, and not as autoimmune disorder, in contrast to long-held views [3].

Specific chemotherapy is recommended for the treatment of Chagas disease applying the general assumption that the earlier the specific treatment is initiated greater are the chances of parasitological cure and clinical benefits to the host [4]. At present, Chagas disease chemotherapy is restricted to two drugs, Benznidazole (Bz) and nifurtimox (Nfx). In Brazil, only Bz has been used to etiological treatment of Chagas disease [5] with higher cure ratio observed during acute, congenital and early chronic Indeterminate Chagas disease [4-6]. Despite the low cure rates observed in most patients submitted to treatment during chronic Chagas disease, several studies have suggested that the Bz-treatment should be still recommended during chronic Chagas disease, since it prevents the disease progression, regardless of lack of complete parasite clearance [7-9]. The mechanism of action of benznidazole is not completely clear. It has been proposed that the BZ-mediated trypanocidal mechanism is effective because the nitro group is reduced by parasite enzymes to produce free radicals, superoxide anions, and hydrogen peroxide [10]. One of the major factors potentially influencing the parasite clearance as well as the morbidity control following the treatment for Chagas’ disease is the cooperative action between the drug effects and the host immunological response [11-14]. However little is known about the changes in the host immune following Bz treatment, specifically during chronic Indeterminate Chagas disease that could synergistically act with the BZ-therapy. The coherent understanding of these changes will certainly support the development of new protocol for Bz therapy, intervention and management of chronic Chagas disease. We believe that the analysis of host immunity status pre- and post-treatment is essential to further elucidate the impact of Bz intervention as well as support the rational development of new trypanosomicidal agents. We have previously shown that early after the end of the Bz therapy, the NK-cells and CD8^+^ T-lymphocytes are important sources of IFN-γ and that IL-10 produced by CD4^+^ T-cells and B lymphocytes are putative key element to modulate the immune response and control tissue damage might inducible by the pro-inflammatory response. In the current investigation we would like to test the hypothesis that the Bz-therapy modulates the immune response inducing a long-lasting impact on the immunological status of treated patients, inducing a similar Type-1 modulated cytokine profile as observed early after the end of the treatment [13,14].

Our findings bring new insights to the hypothesis that later on after the end of Bz-treatment, the patients presented a down-regulated cytokine pattern along with the capacity to build a Type-1 modulated cytokine response, upon *T. cruzi* antigen stimuli, supported by pro-inflammatory cytokine from monocytes, NK and CD8^+^ T-cells, counterbalanced by IL-10 from monocytes.

## Methods

### Study population

This cross-sectional study involved 47 subjects classified into three groups referred as IND – untreated Indeterminate Chagas disease patients (n = 15; 3 males and 12 females; mean age = 42.5 ± 5.5 years, ranging from 35–52 years); INDt – Bz-treated Indeterminate Chagas disease patients (n = 14; 4 males and 10 females; mean age = 44.0 ± 8.7 years, ranging from 33–56 years) and NI – non-infected individuals (n = 18, 7 males and 11 females, mean age = 29.0 ± 9.7 years, ranging from 18–47 years).

Indeterminate Chagas disease was diagnosed by at least two positive results on serological tests (indirect hemagglutination, indirect immunofluorescence, or enzyme-linked immunosorbent assay) and no alteration in the clinical and physical examination, besides normal electrocardiogram, chest X-ray, Holter, and echodopplercardiography profiles. The IND patients were recruited at the Ambulatório de doença de Chagas, Hospital das Clínicas, Universidade Federal de Minas Gerais (Belo Horizonte, Minas Gerais, Brazil).

Benznidazole (N-benzyl-2-nitro-1-imidazolacetamide) treatment was carried out in daily doses of 5 mg/kg of body weight, twice a day for 60 days, according to the Brazilian Ministry of Health [15]. The INDt patients were recruited seven years after the end of etiological treatment at the Ambulatório de doença de Chagas, Hospital das Clínicas, Universidade Federal de Minas Gerais (Belo Horizonte, Minas Gerais, Brazil).

The non-infected subjects showing negative serologic tests for antibodies against *T. cruzi* were contacted at the Centro de Hematologia e Hemoterapia de Minas Gerais (Belo Horizonte, Minas Gerais, Brazil).

All study participants provided written informed consent following the guidelines of Ethics Committee of the Minas Gerais Federal University. The study protocol complied with the regulations 196/1996 of Brazilian National Council on Research in Humans and was also approved by the Ethical Committee of Minas Gerais Federal University under protocol # COEP-ETIC 070/00.

### Blood samples

Five milliliters of whole peripheral blood were collected in EDTA anticoagulant for hemogram analysis. Ten milliliters of heparinized peripheral blood were collect from each participant and used for short-term in vitro culture of whole blood. Heparin whole blood samples were maintained at room temperature for up to 12 hours prior processing.

### *Trypanosoma cruzi* epimastigote antigen preparation

Soluble Epimastigote Antigen (EPI) was prepared from a stationary phase Y strain *T. cruzi* epimastigotes grown in LIT-medium. After the third or fourth in vitro passage, epimastigotes were harvested, washed in 15 mM phosphate-buffered saline (PBS), pH 7.4, and resuspended to 10^8^ cells/mL in 15 mM PBS, pH 7.4. The suspension was rapidly frozen at −70°C and thawed at 37°C three times, with a sonication procedure between each step. The crude lysate was centrifuged (37,000 g) for 90 min and the supernatant collected, dialysed overnight against 15 mM PBS, pH 7.4, sterilized by filtration through a 0.22-μm pore membrane (Filter millex, Milipore Products Division, Billerica, MA, USA) and stored at −70°C until use. The protein content was assayed by the method described by Lowry et al. [16].

### Short-term in vitro culture of whole blood

Short-term cultures in vitro were carried out as previously described by Sathler-Avelar et al., [14]. Briefly, 500μL aliquots of heparinized whole blood samples were transferred into 14 mL polypropylene tubes (Falcon®, BD Pharmingen, San Jose, CA, USA) and incubated in the presence of 500μL of RPMI-1640 (GIBCO, Grand Island, NY, USA) – “Control Culture”; 500μL aliquots of heparinized whole blood samples in the presence of EPI soluble antigen at a final concentration of 20 μg/mL in RPMI-1640 – “Stimulated Culture” or Phorbol 12-Myristate 13-Acetate at 25 ng/mL plus ionomycin at 1 μg/mL (Sigma, St Louis, MO, USA) in RPMI-1640 – “PMA stimulated culture”. The Control and Stimulated cultures were pre-incubated for 1 h at 37°C, 5% CO_2_ in a humidified incubator. Afterward, all cultures were incubated for 4 h in at 37°C, 5% CO_2_ in a humidified incubator in the presence of BFA at 10 μg/mL. Following incubation, all cultures were treated with 100μL of EDTA 2 mM in PBS for 15 min (Sigma, St Louis, MO, USA) in order to block the activation process.

### Immunophenotyping for cell subsets and intracellular cytokines analysis

Following the short term in vitro stimulation, all cultures were washed once with 3 mL of FACS buffer prepared as PBS supplemented with 0.5% bovine serum albumin and 0.1% sodium azide (Sigma, St Louis, MO, USA). Samples were resuspended into 1 mL of FACS buffer. Aliquots of 400μL were immunostained in the dark for 30 min at room temperature with 10μL of pre-diluted TriColor-labelled monoclonal antibodies (mAbs) anti-CD4, CD8, CD14, CD16 or CD19 (Caltag, Burlingame, CA, USA). Following incubation, the erythrocyte lysing and cell fixation procedures were performed by adding 2 mL of FACS lysing solution (BD, San Diego, CA, USA) and incubation for 30 min at room temperature. Membrane-stained leucocytes were permeabilized with 3 mL of FACS perm-buffer for 10 min at room temperature (FACS buffer supplemented with 0.5% saponin) and washed once with 2 mL of FACS buffer. Cell suspension aliquots of 30μL were transferred to 96wells round bottom microplates (Falcon®; BD Pharmingen, San Jose, CA, USA) were stained for 30 min at room temperature, in the dark in the presence of 20μL of PE-labelled anti-cytokine mAbs (IL-12, TNF-α, IFN-γ, IL-4, IL-5 and IL-10). All antibodies were diluted 1:50 in FACS perm-buffer and were purchased from BD Pharmingen (San Diego, CA, USA). After intracytoplasmic cytokine staining, the cells were washed twice with 200μL of FACS perm-buffer and FACS buffer, respectively, and then fixed in 200μL of FACS FIX Solution (10 g/L of paraformaldehyde, 10.2 g/L of sodium cacodilate and 6.63 g/L of sodium chloride, pH 7.2, all from Sigma, St Louis, MO, USA). The samples were stored at 4°C prior flow cytometry acquisition and analysis.

### Flow cytometry acquisition and analysis

Flow cytometry acquisition of 30,000 immunestained cells/samples were performed in a FACScalibur® flow cytometer equipped with CELLQUEST^TM^ software (Becton Dickinson, San Jose, CA, USA). Distinct gating strategies were used to analyze the cytokine-expressing leucocyte subpopulations from the innate (monocytes, neutrophils and NK-cells) and adaptive immunity (lymphocytes as well as T- and B-cell subsets). The monocytes were selected as CD14^High+^ cells and the neutrophil as SSC^High^CD14^Low+^ cells on FL3/anti-CD14-TC versus SSC/laser side-scatter dot plots. The lymphocyte populations were selected on FSC/laser forward-scatter versus SSC/laser side-scatter dot plots. Following the initial gate selection, the frequencies of cytokine^+^ cells were quantified by quadrant statistics applied on FSC/laser forward-scatter versus FL2/anti-cytokine-PE dot plots for monocytes and neutrophils and FL3/anti-CD16, CD4, CD8 or CD19 TC versus FL2/anti-cytokine-PE dot plots for NK-cells, T-lymphocyte subsets and B-cells.

### Analysis of low and high cytokine-producers

The analysis of cytokine-producers within IND and INDt groups were performed by first taking the median value of cytokine^+^ cells from each leucocyte subpopulation (neutrophils, monocytes, NK-cells, CD4^+^ and CD8^+^ T-cells and B-cells) from the NI group as the cut-off edge to categorize the subjects as Low and High cytokine-producers. Gray-scale diagrams were used to assemble the prevalence of Low (empty rectangle), High inflammatory (black rectangle for IL-12, TNF-α and IFN-γ) or High regulatory (gray rectangle for IL-4, IL-5 and IL-10) cytokine-producers amongst IND and INDt groups. Bar charts were applied to compile the frequency of Low and High cytokine-producers within each leucocyte subset.

### Statistical analysis

Statistical analysis was performed using the GraphPad Prism 5 software package (San Diego, CA, USA). As all data files assume a non-Gaussian distribution, statistical comparisons were carried out using the nonparametric Kruskal-Wallis test followed by Dunns multiple comparison test to evaluate the cytokine profiles among NI, IND and INDt groups. The comparative analyses between control culture and stimulated culture were performed by Wilcoxon matched pairs test. The frequency of high and low cytokine-producers was compared by contingency table analysis by Chi-square test. The differences were considered significant when p-values were <0.05.

## Results

### Cytokine profile of innate immunity in indeterminate chagas disease patients

**Figure 1:**
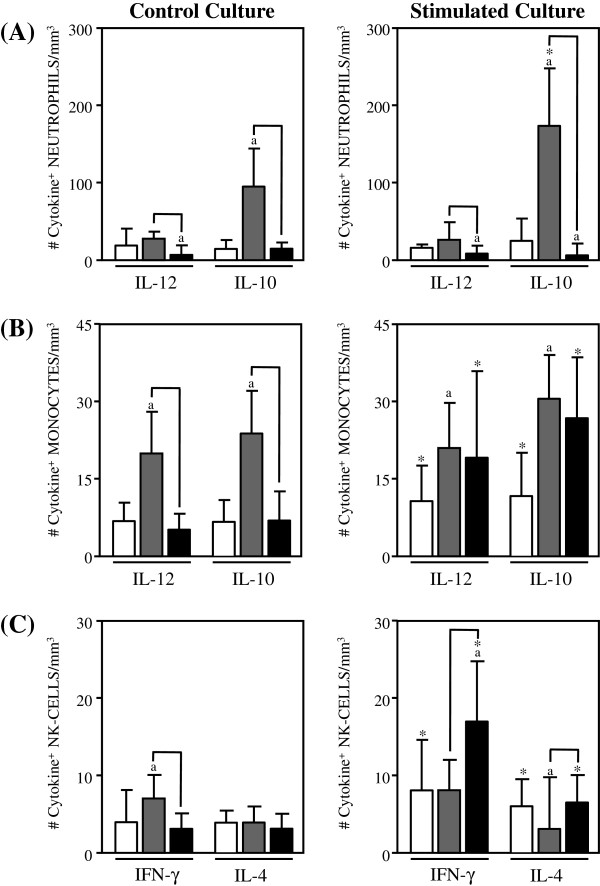
**Analysis of intracytoplasmic cytokine profile of neutrophils. (A)**, monocytes **(B) **and NK-cells in the peripheral blood from non treated Indeterminate chagasic patients (IND gray rectangle), Bz-treated Indeterminate chagasic patients (INDt black rectangle) as compared to non-infected individual (NI empty rectangle). Data are expressed as median absolute counts plus the interquartile range of cytokine^+^ cells observed after short-term *in vitro *“Control Culture (left panels) and *T. cruzi* epimastigote antigen “Stimulated Culture (right panels) of whole-blood samples. Cytokine^+^ neutrophils, monocytes and NK-cells were quantified by dual color immunophenotyping (anti-CD14-TC or anti-CD16-TC plus anti-cytokine-PE mAbs) using specific gate strategies to select each leucocyte subset, as described in Material and Methods. The significant differences at p<0.05 for comparisons with NI are indicated by the letter ‘a. The significant differences at p<0.05 for comparisons with INDt are indicated by connecting lines. The significant differences at p<0.05 for comparative analysis between “Control and “Stimulated cultures are indicated by *.

The number of inflammatory and regulatory cytokines, expressed by the innate immunity cells, from Indeterminate Chagas disease patients is shown in Figure 1.

Data from the control culture showed a prominent anti-inflammatory cytokine pattern in neutrophils from IND as compared to NI, represented by enhanced levels of IL-10^+^ cells and basal levels of IL-12^+^ cells (Figure 1A, left panel). Upon in vitro antigen stimulation, a similar anti-inflammatory cytokine profile was observed in IND, with increased number of IL-10^+^ neutrophils as compared to the NI and control culture (Figure 1A, right panel).

The analyses of monocytes reveled a mixed inflammatory/anti-inflammatory cytokine pattern in IND as compared to NI, with increased levels of IL-12^+^ and IL-10^+^ cells (Figure 1B, left panel). This mixed profile was preserved after in vitro antigen stimulation, as shown by the increased number of IL-12^+^ and IL-10^+^ monocytes in IND as compared to NI (Figure 1B, right panel).

Analysis of NK-cells showed a predominant inflammatory cytokine pattern in IND, with higher number of IFN-γ^+^ cells and basal levels of IL-4^+^ cells as compared to NI (Figure 1C, left panel). Upon in vitro antigen stimulation, despite no difference in the number of IFN-γ^+^ cells, the lower levels of IL-4^+^ cells count to the predominant inflammatory cytokine pattern of NK-cells observed in IND as compared to NI (Figure 1C, right panel).

### Impact of bz-treatment on the cytokine pattern of innate immunity of indeterminate chagas disease patients

In order to investigate the impact of etiological treatment on the cytokine profile of innate immunity of Indeterminate Chagas disease patients, we have compared the cytokine profile of neutrophils, monocytes and NK-cells from INDt as compared to IND. These differences are highlighted by connecting lines.

Data analyses showed an overall cytokine down-regulation in the innate immunity compartment after benznidazole therapy, as shown by the lower levels of IL-12^+^, IL-10^+^ neutrophils and monocytes, as well as reduced levels of IFN-γ^+^ NK-cells in INDt as compared to IND (Figure 1A, B and C, left panel).

However, the in vitro antigen stimulation was able to shift the cytokine expression by monocytes and NK-cells toward a type 1-regulated immune profile, with increased levels of IL-12 and IFN-γ respectively counterbalanced by IL-10 from monocytes (Figure 1B and C, right panel), despite no changes in the cytokine profile of neutrophils (Figure 1A, right panel).

### Cytokine profile of adaptive immunity in indeterminate chagas disease patients

**Figure 2:**
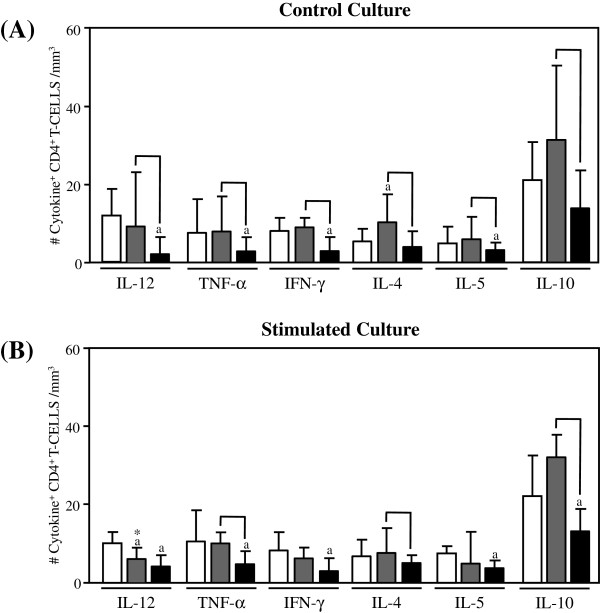
**Analysis of intracytoplasmic cytokine profile of CD4**^**+**^**.** T-lymphocytes in the peripheral blood from untreated Indeterminate chagasic patients (IND gray rectangle), Bz-treated Indeterminate chagasic patients (INDt black rectangle) as compared to non-infected individual (NI empty rectangle). Data are expressed as median absolute counts plus the interquartile range of cytokine^+^ cells observed after short-term *in vitro* “Control Culture” (top panel) and *T. cruzi *epimastigote antigen “Stimulated Culture” (bottom panel) of whole-blood samples. Cytokine^+^ CD4^+^ T-cells were quantified by dual color immunophenotyping (anti-CD4-TC plus anti-cytokine-PE mAbs) using specific gate strategy, as described in Material and Methods. The significant differences at p < 0.05 for comparisons with NI are indicated by the letter ‘a’. The significant differences at p < 0.05 for comparisons with INDt are indicated by connecting lines. The significant differences at p < 0.05 for comparative analysis between “Control” and “Stimulated” cultures are indicated by *.

**Figure 3:**
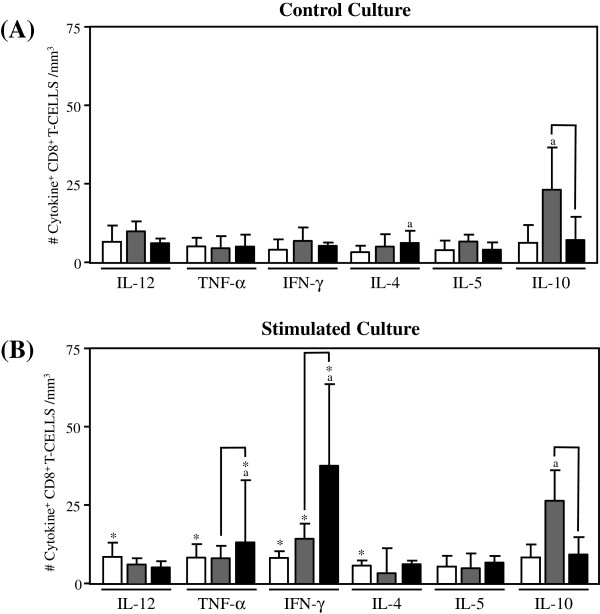
**Analysis of intracytoplasmic cytokine profile of CD8**^**+**^**.** T-lymphocytes in the peripheral blood from untreated Indeterminate chagasic patients (IND gray rectangle), Bz-treated Indeterminate chagasic patients (INDt black rectangle) as compared to non-infected individual (NI empty rectangle). Data are expressed as median absolute counts plus the interquartile range of cytokine^+^ cells observed after short-term *in vitro* “Control Culture” (top panel) and *T. cruzi *epimastigote antigen “Stimulated Culture” (bottom panel) of whole-blood samples. Cytokine^+^ CD8^+^ T-cells were quantified by dual color immunophenotyping (anti-CD8-TC plus anti-cytokine-PE mAbs) using specific gate strategy, as described in Material and Methods. The significant differences at p < 0.05 for comparisons with NI are indicated by the letter ‘a’. The significant differences at p < 0.05 for comparisons with INDt are indicated by connecting lines. The significant differences at p < 0.05 for comparative analysis between “Control” and “Stimulated” cultures are indicated by *.

**Figure 4:**
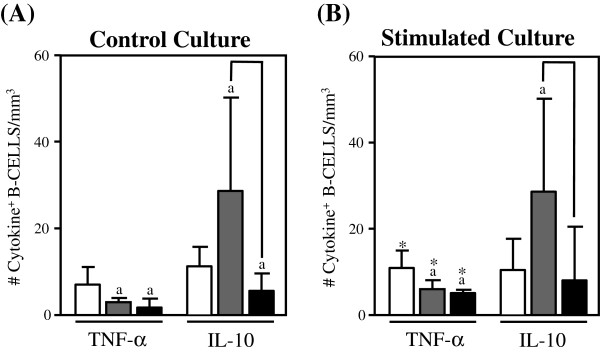
**Analysis of intracytoplasmic cytokine profile of B-cells in the peripheral blood from untreated.** Indeterminate chagasic patients (IND gray rectangle), Bz-treated Indeterminate chagasic patients (INDt black rectangle) as compared to non-infected individual (NI empty rectangle). Data are expressed as median absolute counts plus the interquartile range of cytokine^+^ cells observed after short-term *in vitro* “Control Culture” (left panel) and *T. cruzi* epimastigote antigen “Stimulated Culture” (right panel) of whole-blood samples. Cytokine^+^ B-cells were quantified by dual color immunophenotyping (anti-CD19-TC plus anti-cytokine-PE mAbs) using specific gate strategy, as described in Material and Methods. The significant differences at p < 0.05 for comparisons with NI are indicated by the letter ‘a’. The significant differences at p < 0.05 for comparisons with INDt are indicated by connecting lines. The significant differences at p < 0.05 for comparative analysis between “Control” and “Stimulated” cultures are indicated by *.

The levels of inflammatory and regulatory cytokine-expressing cells in the adaptive immunity compartment from Indeterminate Chagas disease patients are shown in Figures 2, 3 and 4.

Data from the control culture showed a small shift towards a anti-inflammatory cytokine pattern in CD4^+^ T-lymphocytes from IND as compared to NI, represented by a slight increase in the number of IL-4^+^ along with the basal numbers of TNF-α^+^, IL-12^+^, IFN-γ^+^, IL-5^+^ and IL-10^+^ cells (Figure 2A). Upon in vitro antigen stimulation despite the general basal levels of cytokines observed for CD4^+^ T-cells, the small decrease in the IL-12^+^ cells could support the small shift towards a anti-inflammatory cytokine pattern in CD4^+^ T-lymphocytes.

A typical anti-inflammatory cytokine profile was observed for both CD8^+^ T-lymphocytes and B-cells from IND as compared to NI, represented by increased levels of IL-10^+^ cells along with a lower number of TNF-α^+^ B-cells (Figure 3A and 4A). Upon in vitro antigen stimulation, despite the increased number of IFN-γ^+^CD8^+^ T-lymphocytes and TNF-α^+^ B-cells observed in comparison to the control culture, a anti-inflammatory cytokine profile still remain preponderant in both CD8^+^ T-lymphocytes and B-cells from IND as compared to NI (Figures 3B and 4B).

### Impact of bz-treatment on the cytokine pattern of adaptive immunity of indeterminate chagas disease patients

The comparative analysis of cytokine-expressing cells in the adaptive immunity compartment after the etiological treatment is shown in Figures 2, 3 and 4.

Data from the control culture showed an overall cytokine down-regulation in CD4^+^ T-cells from INDt as compared with IND and NI, with decreased levels of IL-12^+^, TNF-α^+^, IFN-γ^+^ and IL-5^+^ cells, along with basal numbers of IL-4^+^ and IL-10^+^ cells (Figure 2A) with no changes observed after the in vitro antigen stimulation (Figure 2B).

On the other hand, despite the basal levels of cytokine-expressing CD8^+^ T-cells observed in the control cultures from INDt as compared to IND (Figure 3A), the in vitro antigen stimulation was able to drive the CD8^+^ T-cells towards a inflammatory cytokine profile, with increased numbers of TNF-α^+^ and IFN-γ^+^CD8^+^ T-cells in INDt as compared to IND and NI (Figure 3B).

Data analysis from the control cultures also showed a cytokine down-regulation in B-cells from INDt as compared with IND and NI, with decreased levels of IL-10^+^ cells (Figure 4A) which remain after in vitro antigen stimulation, regardless of slight increase in TNF-α^+^ B-cells observed in comparison to the control culture (Figure 4B).

### Analysis of low and high cytokine-producers amongst indeterminate chagas disease patients

**Figure 5:**
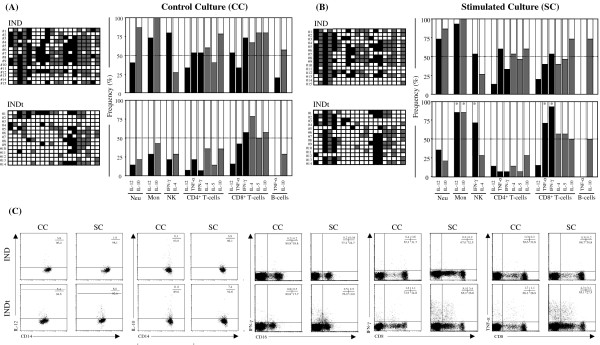
**Frequency of low cytokine-producers (empty rectangle). **High inflammatory cytokine-producers (black rectangle) and high regulatory cytokine-producers (gray rectangle) for each leukocyte subset in the peripheral blood from untreated Indeterminate chagasic patients (IND) and Bz-treated Indeterminate chagasic patients (INDt) using the median percentage of cytokine^+^ cells from the group of non-infected individuals as the cut-off edge. Color diagrams (left panels) were first assembled for individual samples to further compile the resultant frequency (right panels) of cytokine producers in the “Control Culture” (**A**) and *T. cruzi *epimastigote antigen “Stimulated Culture” (**B**) of whole-blood samples. Statistical significance at p < 0.05 for comparisons between “Control” and “Stimulated” cultures are represented by *. (**C**) Representative dot plots illustrating the overall cytokine down-regulation after Bz treatment, as well as the *in vitro *antigen stimulation shifted the cytokine profile toward a type 1-modulated profile.

As previously report by Bahia-Oliveira et al. [17] and Vitelli-Avelar et al. [18], the analysis of Low and High cytokine-producers represents a powerful tool to characterize distinct immunological profiles in Chagas disease patients (Figure 5A and B, top panels). The frequencies of High cytokine-producers in the IND and INDt groups were obtained considering the median of a given cytokine^+^ cells from the NI group as the cut-off edge to categorize the subjects as Low and High cytokine-producers. We have used gray-scale diagrams to assemble the frequency of Low (empty rectangle), High inflammatory (black rectangle) and regulatory (gray rectangle) cytokine-producers amongst IND and INDt groups. Using this concept, our data confirmed the presence of a type-1 modulated cytokine profile within innate immunity cells, with enhanced frequency of High cytokine producers, including IL-10 from neutrophils, IL-12 and IL-10 from monocytes and IFN-γ from NK-cells. The analysis of the adaptive compartment also confirmed the presence of a predominant anti-inflammatory immune response in CD4^+^, CD8^+^ and B-cells and additionally pointed out the presence of High cytokine producers including IL-10 from CD4^+^ T-cells and IFN-γ from CD8^+^ T-cells (Figure 5A top panel).

No differences were observed in the frequency of Low and High producers between control and stimulated cultures from IND (Figure 4A and B, top panel).

### Impact of bz-treatment of the frequency of low and high cytokine-producers amongst indeterminate chagas disease patients

Aiming to further apply the concept of Low and High cytokine producers, we investigate the cytokine profile after Bz-chemotherapy in patients with Indeterminate Chagas disease (Figure 5A and B, bottom panels).

Data analysis further confirmed the broad cytokine down-regulation in both innate and adaptive immunity compartment after Bz-treatment (Figure 5A bottom panel).

Upon in vitro antigen stimulation, the leucocytes from INDt were able to shift the down-regulated cytokine profile toward a type 1 modulated pattern, characterized by enhanced frequency of High cytokine producers including IFN-γ from NK-cells and TNF-α and IFN-γ from CD8^+^ T-cell along with IL-12 and IL-10 from monocytes (Figure 5A and B, bottom panels).

## Discussion

The first insight to use trypanocidal chemotherapeutic drugs in patients with Chagas disease started almost eighty years ago [19]. However, at the present, only Nfx and Bz are clinically available worldwide for the etiological treatment of Chagas disease, with the latter been the main anti-parasitic drug used in Brazil. Despite the effectiveness of Bz to parasite eradication during acute phase of *T. cruzi* infection, the limited efficacy during chronic Chagas disease, besides the high frequency of undesirable side effects have contributed to refuse its use to treat over 8 million patients with chronic Chagas disease [5]. Previous reports have shown that, despite the lack of parasite eradication, the etiological treatment contribute to reduce the parasitemia and rearrange the host immune response, leading to balanced inflammatory response crucial to control Chagas disease morbidity [7,9,13,14,20]. Regardless these insights, there are still few studies focusing on the immunological changes following Bz-treatment during chronic Chagas disease [13,14,21-23]. A coherent understanding of the immunological following Chagas disease etiological treatment will certainly contribute to determine new perspectives for immunoprevention, therapy, intervention and management [24,25]. In this context, it is important to mention that the development of longitudinal studies would be the choice to avoid possible experimental bias due to particular features of the individual immune response. However, longitudinal interventions presents besides the difficulty to establish long-term follow-up of patients, a large gap between the first and the second evaluation, especially when focusing on long-lasting features, that would introduce a relevant variable to the experimental approaches, mainly due to the reagent lots and equipment performances.

In this study, we have performed a cross-sectional investigation to verify whether the Bz-therapy would induce a long-lasting impact on the immunological status of treated patients leading to a Type-1 modulated cytokine profile likely that observed early after the end of the treatment [13,14]. For this purpose, we have first characterized the cytokine profile of circulating leucocytes from untreated IND patients in order to establish a baseline to evaluate the impact of etiological treatment during chronic *T. cruzi* infection.

Our data showed that untreated IND patients presented in the ex vivo analysis (Control Culture) an overall type-1 regulated cytokine profile in the innate immune compartment (IL-10 from neutrophils, IL-12 and IL-10 from monocytes and IFN-γ from NK-cells) and a predominant type-2 profile in the adaptive immunity cells (IL-4 from CD4^+^ T-cells, IL-10 from CD8^+^ T-cells and B-lymphocytes). It was interesting to notice that the cytokine profile of innate and adaptive immunity cells was generally preserved upon in vitro antigenic stimulation.

These findings showed that untreated IND patients are able to mount a pro-inflammatory cytokine response supported by enhanced levels of IL-12^+^ monocytes and IFN-γ^+^ NK-cells. However, this inflammatory response seems to be regulated in Indeterminate clinical form, to consequently prevent the disease progression, by the establishment of modulatory mechanisms that involve the participation of IL-10 from neutrophils, monocytes, CD8^+^ T-cells and B-cells. Our findings showed an increased frequency of inflammatory-like monocytes and NK-cells, together with high levels of regulatory-like monocytes and modulated adaptive immunity that could be important to the establishment/maintenance of asymptomatic chronic Chagas disease. Previous studies have shown that the establishment of an inflammatory immune response mediated by monocytes and NK-cells are essential to control *T. cruzi* infection [26,27] and that the ability to mount immunoregulatory mechanisms in both innate [28] and adaptive immunity [19] are essential to control the inflammatory immune activity and are apparently necessary to prevent a deleterious effect of anti-*T. cruzi* immune response during asymptomatic chronic Chagas disease.

The analysis of High and Low cytokine producers has been proposed by Bahia-Oliveira et al. [17] and further applied by Vitelli-Avelar et al. [18] to evaluate the cytokine profile of Chagas disease patients. Using this approach, our data further confirmed the cytokine profile described above. Additionally, our findings pointed out the presence of increased frequency of High IL-10 producers within CD4^+^ T-cells, but still lower than the frequency of High IL-10 producers within monocytes. These data re-inforce previous reports form Gomes et al., [28] demonstrating that in IND the majority of the IL-10-producing cells are monocytes. Moreover, our data showed that enhanced frequency of High IFN-γ producers could be identified within CD8^+^ T-cells in IND, not detectable by the conventional analysis. This finding is also in agreement with those presented by Gomes et al. [28] showing that around 60% of the IND could be identified as High IFN-γ producers. These authors suggested that the IND patients that are high IFN-γ producers are candidates to develop cardiomyopathy sooner than those lower producers. On the other hand, it is possible that IND patients who are able to always keep high levels of IL-10 secretion has less chance to develop cardiac disease [28]. Human longitudinal studies under evaluation by our group will be able to validate this hypothesis.

As IND is not necessarily a permanent clinical form, with approximately 36% of them eventually developing severe cardiac disease [1], any effort that could make to maintain the IND clinical status, apart from parasitological cure, would be relevant to control Chagas disease morbidity. It has been suggested that the etiological treatment could be employed to maintain the stable Indeterminate clinical form and prevent the disease progression, despite the parasitological cure [7-9,21]. Since impairment in the cytokine network has been pointed out as one of the determining factors driving disease morbidity [2,18], it is possible that if the etiological treatment would be able to introduce changes in the cytokine network towards immunodulatory mechanisms, it could contribute to prevent the disease progression. In this study, we have characterized the cytokine profile of circulating leucocytes from treated INDt patients, seven years after the end of Bz chemotherapy and compared with that observed in untreated IND patients.

Our data showed that treated INDt patients presented, in the ex vivo analysis (Control Culture), an overall down-regulated cytokine profile in both, innate immunity (IL-12 and IL-10 from neutrophils and monocytes besides IFN-γ from NK-cells) and adaptive immune compartment (IL-12, TNF-α, IFN-γ and IL-5 from CD4^+^ T-cells and IL-10 from B-cells), along with basal levels of cytokine-expressing cells within CD8^+^ T-lymphocytes. The overall down-regulation in the cytokine synthesis observed seven years after the end of Bz-treatment is in agreement with previous reports the immunomodulatory effect of Bz in experimental models [21,29,30]. This modulated profile may account for the general hypothesis that the Bz-treatment during chronic Chagas disease may has a supportive impact, modulating the inflammatory cytokine profile of peripheral blood leucocytes from IND reducing the chances of progression to cardiac disease [13,14,21]. However, this down-regulation in the cytokine profile may shortly favor the parasite replication from residual inflammatory foci, considering the low effectiveness of Bz-treatment to promote parasite clearance during chronic Chagas disease [31-33]. To investigate the possible impact that residual *T. cruzi* antigens would have on the immune response of Bz-treated INDt patients, we have characterized the cytokine pattern triggered upon in vitro *T. cruzi* antigen stimulation. Interestingly, our data showed that the cytokine profile of innate and adaptive immunity cells was greatly impacted by the antigenic stimulation. In fact, the in vitro antigen stimulation was able to emerge the cytokine synthesis mainly in monocytes (IL-12 and IL-10), NK-cells (IFN-γ) and CD8^+^ T-cells (IFN-γ), characterizing an overall type 1-modulated immune profile. The analysis of High and Low cytokine producers further confirmed the impact of *T. cruzi* antigens on the cytokine pattern of INDt. This modulated pro-inflammatory cytokine pattern resemble that observed in children treated during early Indeterminate Chagas disease (E-INDt), by the ability of NK-cells and CD8^+^ T-cells to produce IFN-γ [14]. However, there was a major difference regarding the source of modulatory IL-10 in INDt (monocytes) and E-INDt (CD4^+^ T-cells and B-lymphocytes) [14].

The ability of the immune system to generate stable memory CD8^+^ T-cell after pathogen clearance during acute and chronic infection have been already shown in experimental *T. cruzi* infection [21,34]. These authors have shown that Bz-induced cure drives conversion of the immune response to a stable and protective CD8^+^ T-cell central memory response, capable of showing effector function and providing protective immunity upon re-challenge [21,34]. In agreement with our data, the Bz-treatment does not result in the full retention of CD4^+^ T-cells able to recall cytokine production upon antigenic stimulation [21]. It has been shown that Bz-treatment of infected hosts leads to a selective expansion of effector and memory CD8^+^ T-cells able to protect the hosts against re-infection. These findings allow these authors to hypothesize that besides the direct role in blocking the parasite replication in vivo, Bz-treatment appears to positively affect the antigen-driven host immune system and thus maintain the participation of immuneprotective effector mechanism in the anti-pathogen response. Therefore, the effectiveness of any chemotherapy protocols should consider not only the trypanocidal effect but also its impact on the host immune response.

Additional studies are still needed to better understand the impact of Bz-treatment on the immune system of patients at distinct clinical forms of Chagas disease. As previously reported, we are currently investigating the impact of Bz-treatment on the immunological status on cardiac Chagas disease patients [14]. Preliminary data have pointed out the beneficial impact of Bz-treatment in the immunological profile in cardiac patients increasing the frequency of IL-10^+^ monocytes, an important immunomodulatory event to control deleterious anti-parasite immune-mediated inflammatory mechanisms [18]. The ability of Bz to induce the persistence of antigen-specific memory CD8^+^ T-cells in cardiac patients are currently under evaluation by our group.

## Conclusion

In summary, our findings showed that the Bz treatment of Indeterminate Chagas’ disease patients shifts the cytokine patterns of peripheral blood monocytes, NK-cells and CD8^+^ T-cells towards a long-lasting Type-1-modulated profile that could be important to the maintenance of a non-deleterious immunological microenvironment.

## Competing interests

The authors have no competing interests to declare.

## Authors’ contributions

SMES and EDG were responsible for the medical screening of patients. OAMF, RSA, DMVA and ATC were involved in the design, acquisition data and analysis. All authors contributed to the interpretation data. OAMF, RSA, DMVA, SMES and ATC drafted the manuscript and all authors revised the final draft critically for important critical content. All authors have given final approval of the version to be published

## Pre-publication history

The pre-publication history for this paper can be accessed here:

http://www.biomedcentral.com/1471-2334/12/123/prepub
